# Hierarchical MEC Servers Deployment and User-MEC Server Association in C-RANs over WDM Ring Networks

**DOI:** 10.3390/s20051282

**Published:** 2020-02-27

**Authors:** Zhen Liu, Jiawei Zhang, Yanan Li, Yuefeng Ji

**Affiliations:** State Key Lab of Information Photonics and Optical Communications, Beijing University of Posts and Telecommunications, Beijing 100876, China; liuzhen207@bupt.edu.cn (Z.L.); zjw@bupt.edu.cn (J.Z.); liyn@bupt.edu.cn (Y.L.)

**Keywords:** mobile edge computing (MEC), cloud radio access network (C-RAN), hierarchical deployment of MEC servers, user allocation

## Abstract

With the increasing number of Internet of Things (IoT) devices, a huge amount of latency-sensitive and computation-intensive IoT applications have been injected into the network. Deploying mobile edge computing (MEC) servers in cloud radio access network (C-RAN) is a promising candidate, which brings a number of critical IoT applications to the edge network, to reduce the heavy traffic load and the end-to-end latency. The MEC server’s deployment mechanism is highly related to the user allocation. Therefore, in this paper, we study hierarchical deployment of MEC servers and user allocation problem. We first formulate the problem as a mixed integer nonlinear programming (MINLP) model to minimize the deployment cost and average latency. In terms of the MINLP model, we then propose an enumeration algorithm and approximate algorithm based on the improved entropy weight and TOPSIS methods. Numerical results show that the proposed algorithms can reduce the total cost, and the approximate algorithm has lower total cost comparing the heaviest-location first and the latency-based algorithms.

## 1. Introduction

Internet of Things (IoT) enables billions of sensors, devices, and actuators, as well as the human to be interconnected via the Internet over a distributed environment to work together. The European Commission has predicted that there will be 50 to 100 billion devices connected to the Internet by 2020 [1]. The fast increase of mobile data traffic generated by IoT devices, and the stringent requirements of the emerging applications in terms of latency and bandwidth, have spurred numerous influences for the evolution of cloud radio access network (C-RAN) [2,3] and mobile cloud computing (MCC) [4]. On the one hand, in the original C-RAN with baseband unit (BBU) [5], since BBU is far from IoT devices, it is difficult to meet the requirements of latency-sensitive applications. Therefore, C-RAN with central units and distributed units is proposed by the latest 3GPP technical reports [6] to improve the flexibility of the network and thus to meet the differentiated demands of IoT applications. On the other hand, MCC requires transporting data to core datacenters (DCs) over the core network, which faces challenges in terms of latency and bandwidth [7]. For these issues, mobile edge computing (MEC) is introduced by the European Telecommunications Standards Institute (ETSI) [8], whose aim is to provide computing capabilities in close proximity to IoT devices for enhanced service/application performance. Therefore, MEC enabled C-RAN has emerged as a promising candidate for the next generation access network techniques in order to offer a service environment characterized by proximity, low latency, and high rate access [9,10].

**Figure 1:**
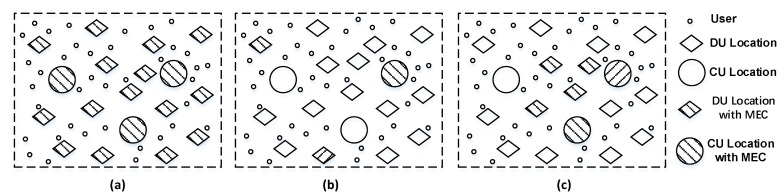
Different deployment solutions of mobile edge computing (MEC) servers in cloud radio access network (C-RAN) (**a**) Full deployment solution of MEC servers. (**b**) Low-cost decentralized deployment solution of MEC servers. (**c**) Centralized deployment solution of MEC servers.

In spite of the promising benefits brought by deploying MEC servers in C-RAN, a large number of users with various requirements pose new challenges. First, due to the limited computing and storage capacity of a single MEC server, a large number of MEC servers need to be deployed in order to meet the quality of service (QoS) of the fast-growing IoT traffic. However, considering the deployment cost constraints, it is unrealistic to deploy the MEC server at each access point [11]. Therefore, how to deploy MEC servers to achieve a tradeoff between users’ QoS and deployment cost is a challenge. Second, when there are multiple user demands to be responded to in the C-RAN, we need to consider which users to assign to which MEC servers. This is because the user assignment among MEC servers will impact the users’ QoS. When the workload of an MEC server is too heavy, the computing latency of the user is increased, resulting in the intolerant response delay. Therefore, how to assign the users among MEC servers is challengeable. Third, massive data are pushed into the network, which imposes further pressure in the access network. The optical access networks based on wavelength division multiplexing (WDM) with low-cost, low latency, and high capacity are considered as an outstanding candidate [12,13,14]. Thus, assigning the routing and wavelength for data in C-RAN over WDM networks is also a key issue. Note that the challenges mentioned above are closely related to the deployment of MEC servers. The deployment scheme will directly affect users’ QoS, economic benefits of operators, and network performance. Figure 1 shows three examples of MEC servers’ deployment in C-RAN. As shown in Figure 1a, the MEC servers are deployed at each candidate location, which guarantees the users’ QoS (e.g., latency and capacity requirement) but at the expense of deployment cost. As shown in Figure 1b, in order to reduce deployment cost, a small number of MEC servers are deployed, which results in an increase in the number of users in each MEC server, thus increasing the queuing latency of users in the MEC server. In Figure 1c, the MEC servers gather around in the same area, which needs to route data through the congested nodes, resulting in severe network congestion and an increase in network latency of users. Therefore, poor deployment of MEC servers may lead to the degradation of users’ QoS and operators’ economic benefit.

**Figure 2:**
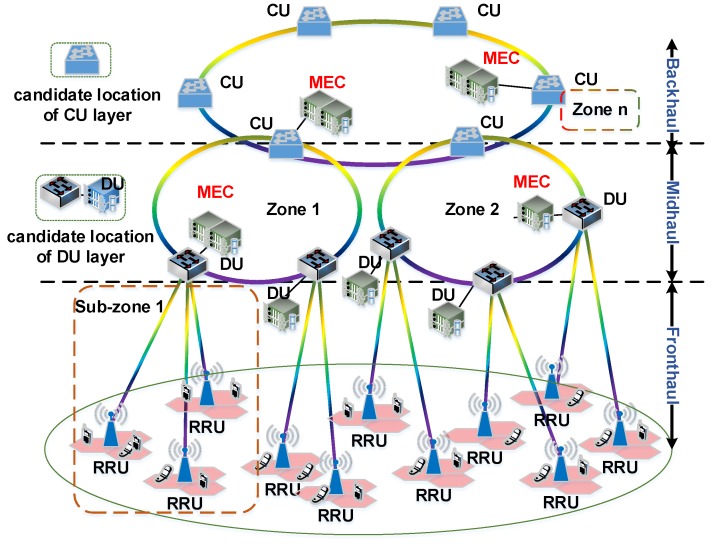
Three-tier C-RAN architecture supporting MEC servers’ deployment.

In this paper, we leverage the C-RAN architecture (Figure 2) and propose a hierarchical deployment scheme to jointly optimize MEC servers’ deployment and request allocation with the objective to minimize the deployment cost and average latency. In particular, the scheme deploys relatively large MEC servers at the central units and smaller MEC servers at the distributed units. We focus on solving the following optimization problems: (1) in which locations MEC servers should be deployed; (2) how many MEC servers should be deployed; (3) which requests should be assigned to which MEC servers. The key contributions of this study are as follows:

(i) We present a hierarchical architecture for MEC servers’ deployment and requests allocation based on C-RAN, where MEC servers can be deployed at the distributed units or at the central units.
(ii) We cast a mathematical framework to investigate the average latency-deployment cost tradeoff problem by associating with computation and communication resource allocation. The average latency of requests includes network latency and computing latency: network latency depends on the length of the optimal path selected between the location of request and MEC server by using the routing and wavelength assignment scheme and computing latency is modeled as an M/M/1 queuing model.
(iii) We propose an enumeration algorithm for MEC servers deployment and request allocation as a benchmark, which enumerates all possible deployment sets. The requests allocation scheme considers the master-slave characteristic of candidate locations at central unit (CU) and distributed unit. In order to find the deployment set for MEC servers in a reasonable time scale, we propose an approximate algorithm for solving the MEC servers’ deployment problem effectively, which combines entropy weight method and TOPSIS method based on unassigned requests ratio.

The rest of this paper is organized as follows. Section 2 provides a comparison with existing solutions to highlight the novelties of the proposed research. In Section 3, we introduce the system model. In Section 4, a mathematical model of MEC servers deployment problem is presented. In Section 5, two heuristic algorithms of MEC servers deployment are discussed. We present the simulation results in Section 6. Section 7 concludes the paper.

## 2. Related Works

The MEC servers/cloudlets deployment and requests allocation are of great importance in networks, which have been attracting more attention in recent years [15]. The latency [16,17,18,19] and deployment cost [20,21] are usually considered as the criterions for performance evaluation.

In the respect to latency, Xu et al. [16] studied cloudlet placement problem that deployed K capacitated cloudlets with the objective of minimizing the average network latency between mobile users and their cloudlets. Zhao et al. [17] investigated the cloudlets deployment problem to minimize average access delay with considering the queuing process. Jia et al. [18] proposed an algorithm to deploy K cloudlets and assigned the users workload among the cloudlets, which reduces the sum of network latency and computing latency. Wong et al. [19] proposed to install cloudlets within the optical access network to meet stringent latency requirements. However, these works only consider the latency of users, without considering the deployment cost.

In the respect of deployment cost, Mondal et al. [20] proposed a hybrid cloudlet placement framework based on TDM-POM access, which includes three tiers placement locations (field, remote node, central office). A nonlinear mixed-integer program was established based on hybrid cloudlet placement framework to minimize the deployment cost. Ma et al. [21] studied the cloudlet deployment and resource allocation to minimize the number of cloudlets.

There are some authors focusing on the tradeoff between latency and deployment cost. Fan et al. [22] proposed the placement scheme of deploying K cloudlets at base stations to minimize the deployment cost and latency. However, in [22] only consider deploying cloudlets at the base station level, which results in high capital expenditure and operational expenditure due to limited capacity at the base station s. To compensate for the relatively small capacity at the base station s, our previous work [23] presented a MEC servers hierarchical deployment framework to deploy MEC servers at the base station level and metro level. Based on the hierarchical deployment framework, a mathematical model is proposed to minimize the deployment cost and E2E latency. While it is possible to implement relatively large capacity at the metro level, obtaining services from MEC servers located on the metro side still undertakes considerable latency and bandwidth due to the relatively long distance between users and MEC servers of metro side.

In this paper, we propose a hierarchical deployment scheme based on C-RAN to exploit the tradeoff between deployment cost and average latency by jointly optimizing MEC servers’ deployment, requests allocation, and routing and wavelength assignment. Instead of regarding the latency as the length of the shortest path, we investigate the latency issue from a much more detailed perspective, i.e., the network latency takes into account available wavelength of links, and the computing latency is modeled as an M/M/1 queuing model.

## 3. System Model

In this section, a three-tier C-RAN architecture supporting hierarchical deployment of MEC servers is given, and the network model of MEC servers deployment is elaborated.

### 3.1. Introduction to Cloud Radio Access Network (C-RAN) and Mobile Edge Computing (MEC)

C-RAN: In C-RAN, the original BaseBand Unit (BBU) and Remote Radio Units (RRU) are reconstructed as three functional entities in 5G. A typical three-tier C-RAN, as shown in Figure 2, is constituted of: (i) light-weight distributed RRU deployed at the cell site, (ii) distributed unit located close to the antenna in the antenna mast and close to the users, (iii) central unit located in centralized locations with high processing capacity [6]. The central unit provides the non-real-time functionalities while the distributed unit provides physical layer functions and the real-time Hard Automatic Repeat reQuest (HARQ) and Automatic Repeat reQuest (ARQ) ones [24]. The network segment between distributed unit and RRU is the new fronthaul, where a distributed unit services multiple RRUs and each RRU is served from a single distributed unit. The network segment between the distributed unit and central unit is referred as mid-haul, where central unit services multiple distributed units and each distributed unit is served from a single central unit. Moreover, multiple central units are interconnected in the backhaul.

MEC: MEC as an alternative for resource-intensive and latency-intolerant applications, which is considered to be an extension of the cloud computing paradigm to edge networks [7]. MEC servers offer cloud-computing capacity within RAN in close proximity to IoT devices. In the paradigm of MEC, requests are served by nearby edge nodes, which can reduce the bandwidth consumption of the core network and minimize the end-to-end latency. Owing to the limited computing and storage capacities of MEC server, there would be lots of MEC servers distributed geographically in C-RAN.

### 3.2. C-RAN Based MEC Servers Deployment

Figure 2 illustrates the three-tier C-RAN architecture supporting MEC servers’ deployment. We consider an optical access networks based on WDM where nodes are assumed as hierarchically organized into a three-stages “ring-and-spur” architecture. In WDM ring network, the adjacent nodes are connected by a pair of fibers forming a loop structure. Each link is equipped with multiple fibers, and each fiber contains a number of wavelengths. In this architecture, MEC servers can be deployed either in the distributed unit (DU) or central unit (CU) locations, so that close proximity with the IoT devices is maintained [25].

For the MEC servers at DU layer, point-to-point fiber links between a MEC server and each RRU connected to it are installed. Because MEC servers of DU layer are in close proximity with the IoT devices, the network latency and bandwidth consumption are lower. However, the coverage of MEC servers at DU layer is limited, and the cost is high. The idea of installing MEC servers at central unit layer can serve a large number of users, and has a lower cost. However, the network latency and bandwidth consumption increases more in MEC servers at DU layer [26].

Therefore, MEC servers at central unit layer are more economical option over MEC servers at DU layer, especially in dense deployment scenarios. Compared with the MEC server deployed at DU layer, MEC server deployed at the central unit layer has more computing and storage capacity. However, the network latency and bandwidth are the key drawbacks, due to the relatively long distance between MEC servers and users.

### 3.3. Network Model

We define *I* = { 1,2,…,*i*,…} as the set of requests, *R* = { 1,2,…,*r*,…} as the set of RRUs, *N* = { 1,2,…,*n*,…} as the set of DUs, *M* = { 1,2,…,*m*,…} as the set of central unit s, and *J* = *N* + *M* = { 1,2,…,*j*,…} as the set of candidate locations of MEC servers. *W* denotes the number of wavelengths of each fiber link in mid-haul and backhaul network. The network is shared by multiple requests. A request *i* is defined as (*d_i_*, *λ_i_*), where *d_i_* is the computing resource demand of request *i*, and *λ_i_* is the average generation rate of request *i.* A candidate location *j* of MEC server is defined as (*f_j_*, *SC_j_*), where *f_j_* is the cost of rental site of location *j, SC_j_* is the number of physical machines of location *j*. The detailed notations and definitions used in this paper are summarized in Table 1.

## 4. Problem Definition and Formulation

In this section, for the MEC servers’ deployment problem, we defined a mathematical model. We assume that we have already known the locations of the RRU, DU, and CU, irrespective of the deployment scenario.

### 4.1. Problem Definition

#### 4.1.1. Computing Latency in MEC Server

Although a MEC server contains a finite set of physical machines, we consider each MEC server as one entity to handle the requests. As the requests arrive in the corresponding optimal MEC server, each request would be assigned an amount of computing resource. Thus, we model the processing of requests in each MEC server as a queuing model and assume the request *i* follows a Poisson distribution with the average generation rate of request equal to *λ_i_* [27,28]. The total incoming service request rate to MEC server *j* is calculated by adding all the service requests arriving associated with MEC server *j*, which is denoted by *∑_i_**λ_i_**•y_i,j_*. Meanwhile, the service time of MEC server *j* for executing requests assigned to it is assumed to be exponentially distributed with the average service time equal to 1/*u_j_*, where *u_j_* is the average service rate of MEC server *j*. Hence, we model the processing of requests by a MEC server as an M/M/1 queueing model [29] and the average computing latency of request *i* in MEC server *j* equal to 1/( *u_j_*-*∑_i_**λ_i_**•y_i,j_*.). Consequently, the average computing latency of request *i* is expressed as follows:

(1) $$\sum_{j\in J}\frac{1}{u_{j}-\sum_{i}\lambda_{i}\cdot y_{i,j}}\cdot y_{i,j}.$$

#### 4.1.2. Network Latency

When a request is sent to a MEC server, the network latency of request comprises: (1) the wireless transmission latency of request from the user to its associated RRU; (2) the network latency for transmitting requests from request’s RRU to destination MEC server. As we can see, different locations of MEC servers will not affect the values of wireless transmission latency. Thus, we will not consider the wireless transmission latency from the user to its associated RRU. The network latency from request’s RRU to destination MEC server depends on the length of a path between the location of request and MEC server. Thus, the network latency between request *i* and its potential MEC server *j* can be expressed as

(2) $$\nu \cdot [\sum_{n\in N}\sum_{r\in R}Z_{i,r}\cdot Z_{r,n}\cdot d_{\left(r,n\right)}\cdot y_{i,n}+\sum_{j\in J/n}(\sum_{n\in N}\sum_{r\in R}Z_{i,r}\cdot Z_{r,n}\cdot d_{\left(r,n\right)}+\sum_{n\in N}\sum_{l,m\in J}Q_{i,n,j}^{(l,m),w}\cdot Z_{i,n}\cdot d_{\left(l,m\right)})\cdot y_{i,j}]$$

There are three items in the square brackets of (2). The first one denotes the network latency of request *i* if request *i* within the coverage area of DU *n* is handled by MEC server located at local DU *n*. The second and third items denote the network latency of request *i* if request *i* is handled by MEC server located at non-local DU *n*.

#### 4.1.3. Deployment Cost of Unit Workload

When providers deploy MEC servers, they not only choose locations for MEC servers, but also determine the optimal number of deployed MEC servers. The deployment cost includes two parts: the cost of the rental site and the cost of the basic equipment. Among them, the cost of rental site depends on the location of the MEC server and the cost of the basic equipment depends on the number of physical machines in an MEC server.

In this paper, the MEC servers can be installed either in DU layer or CU layer. The MEC servers deployed at CU layer is more economical option over MEC servers at DU layer. This is because the capacity of the MEC servers at CU layer is large than that of the MEC servers at DU layer. For unit workload, the rental site cost of the MEC server at CU layer is lower than the rental site cost of the MEC server at DU layer, while the basic equipment cost of the MEC server at CU layer is the same as the basic equipment cost of the MEC server at DU layer. Therefore, the deployment cost of unit workload at CU layer is lower than the deployment cost of unit workload at DU layer. We consider the deployment cost of unit workload to represent the economic benefit of each candidate location. The deployment cost of unit workload in the network can be expressed as

(3) $$\sum_{j\in J}\frac{f_{j}+P\cdot SC_{j}}{C\cdot SC_{j}}\cdot x_{j}.$$

### 4.2. Problem Formulation

#### 4.2.1. Objective Function

(4) $$\begin{array}{l} \begin{array}{ccc} & & \min\sum_{j\in J}\frac{f_{j}+P\cdot SC_{j}}{C\cdot SC_{j}}\cdot x_{j}+\Psi \cdot \frac{1}{\left|I\right|}\sum_{i\in I}\{ \end{array}[\nu \cdot \sum_{n\in N}\sum_{r\in R}Z_{i,r}\cdot Z_{r,n}\cdot d_{\left(r,n\right)}+\frac{1}{u_{n}-\sum_{i}\lambda_{i}\cdot y_{i,n}}]\cdot y_{i,n}+\\ {}[\sum_{j\in J/n}(\nu \cdot \sum_{n\in N}\sum_{r\in R}Z_{i,r}\cdot Z_{r,n}\cdot d_{\left(r,n\right)}+\nu \cdot \sum_{n\in N}\sum_{l,m\in J}Q_{i,n,j}^{(l,m),w}\cdot Z_{i,n}\cdot d_{\left(l,m\right)}+\frac{1}{u_{j}-\sum_{i}\lambda_{i}\cdot y_{i,j}})\cdot y_{i,j}]\} \end{array}$$

Here, the first term denotes the deployment cost. The second and third terms denote the sum of network latency and computing latency of request *i* if request *i* within the coverage area of DU *n* is handled by MEC server located at local DU *n*. The fourth, fifth, and sixth terms denote the sum of network latency and computing latency of request *i* if request *i* is handled by MEC server located at non-local DU *n*.

#### 4.2.2. Constraints

(1) Uniqueness constraint for user assignment:

(5) $$C1:\begin{array}{cc} & \sum_{j\in J}y_{i,j}=1,\begin{array}{cc} & \forall i \end{array}\in I \end{array}$$

C1 is to ensure that each request is assigned to only one MEC server.

(2) Relationship between *y_i,j_* and *x_i_*:

(6) $$C2:\begin{array}{cc} & y_{i,j}\leq x_{j},\begin{array}{cc} & \forall i \end{array}\in I,\forall j\in J \end{array}$$

C2 ensures that whenever a request is assigned to location *j*, then a MEC server must be deployed to location *j*.

(3) Computing capacity constraint:

(7) $$C3:\begin{array}{cc} & \sum_{i\in I}d_{i}\cdot y_{i,j}\leq C\cdot SC_{j},\begin{array}{cc} & \forall j \end{array}\in J \end{array}$$

C3 imposes the workload of a MEC server not to be more than the capacity of the MEC server.

(4) System stable constraint:

(8) $$C4:\begin{array}{cc} & u_{j}-\sum_{i\in I}\lambda_{i}\cdot y_{i,j}>0,\begin{array}{cc} & \forall j \end{array}\in J \end{array}$$

C4 is to guarantee the average service rate to be more than the average arrival rate for each MEC.

(5) Wavelength uniqueness constraint:

(9) $$C5:\begin{array}{cc} & \sum_{j\in J}\sum_{w\in W}\sum_{n\in N}Q_{i,j}^{w}\cdot Z_{i,n}=1,\begin{array}{cc} & \forall i\in I \end{array} \end{array}$$

C5 indicates that any request can use only one wavelength.

(6) Wavelength continuity constraint:

(10) $$C6:\begin{array}{cc} & Q_{i,j}^{(l,m),w}\leq Q_{i,j}^{w},\begin{array}{cc} & \forall i\in I,\forall j\in J,\forall (l,m)\in E,\forall w\in W \end{array} \end{array}$$

C6 indicates that if the *i*th request passes through link (*i,j*) use wavelength *w*, the *i*th request only use wavelength *w* on all links.

(7) Wavelength clash constraint:

(11) $$C7:\begin{array}{cc} & \sum_{j\in J}\sum_{i\in I}\sum_{n\in N}Q_{i,n,j}^{(l,m),w}\cdot Z_{i,n}\leq 1,\begin{array}{cc} & \forall (l,m)\in E,\forall w\in W \end{array} \end{array}$$

C7 ensures that different requests passing through the same link do not use the same wavelength.

(8) Wavelength capacity constraint:

(12) $$C8:\begin{array}{cc} & \sum_{j\in J}\sum_{i\in I}\sum_{w\in W}\sum_{n\in N}Q_{i,n,j}^{(l,m),w}\cdot Z_{i,n}\leq W,\begin{array}{cc} & \forall (l,m)\in E \end{array} \end{array}$$

(9) Relationship between $Q_{i,j}^{w}$ and *y_i,j_*:

(13) $$C9:\begin{array}{cc} & Q_{i,j}^{w}\leq y_{i,j},\begin{array}{cc} & \forall i\in I,\forall j\in J,\forall w\in W \end{array} \end{array}$$

(10) Flow conservation constraint:

(14) $$C10:\begin{array}{cc} & \sum_{l\in J}\sum_{n\in N}(Q_{i,n,j}^{(l,m),w}-Q_{i,n,j}^{(m,l),w})\cdot Z_{i,n}=\left\{ \begin{array}{l} -Q_{i,j}^{w},\begin{array}{cc} if & m=s_{i} \end{array}\\ Q_{i,j}^{w},\begin{array}{cc} if & m=j \end{array}\\ 0,\begin{array}{cc} if & m\neq s_{i},m\neq j \end{array} \end{array}\right.,\forall i\in I,\forall j\in J,\forall w\in W,\forall m\in J \end{array}$$

C10 guarantees that a request uses a path from the source node and the destination node.

(11) Relationship between latency and deployment cost

(15) $$C11:\begin{array}{cc} & \Psi =\frac{\sum_{j\in J}\left(\frac{f_{j}+P\cdot SC_{j}}{C_{j}}\right)}{\max(t_{i})}\cdot \frac{\eta_{1}}{\eta_{2}} \end{array}$$

C11 is the cost coefficient Ψ, which maps the E2E latency to latency cost. *max(t_i_)* is the maximum E2E latency of request *i*. *η*_1_ and *η*_2_ are tradeoff coefficients of latency cost and deployment cost, respectively, where *η*_1_ + *η*_2_ = 1.

## 5. The Heuristic Algorithm for MEC Servers Deployment and User Allocation

In this section, we devise two algorithms for the MEC servers’ deployment and requests allocation to minimize the sum of deployment cost and latency cost. The algorithms are implemented based on Java on a computer with 3.4GHz, 8.00 GB RAM, and 64 bit operating system.

### 5.1. Enumeration Algorithm

In this solution, we enumerate all possible deployment sets of MEC servers, and compare the total cost among all deployment sets to get the optimal deployment set with minimal total cost. As shown in algorithm 1, we first find all feasible candidate deployment sets. Then, the deployment cost of unit workload and an average latency of all requests are calculated under each feasible candidate deployment set. The main processes are shown in the following steps.

**Step 1**: Find feasible deployment sets for MEC servers. To reduce the computational complexity of algorithm 1, we first calculate the lower limit of the number of deployed MEC servers based on the computing capacity constraint. The lower limit of the number of MEC servers deployed is calculated according to Equation (16) (line 2 in algorithm 1). Then, we use a recursive algorithm to find all candidate deployment sets under different numbers of MEC servers (line 4 in algorithm 1). Finally, we set the candidate deployment sets that the satisfy capacity requirements of all requests as feasible deployment sets (lines 5-9 in Algorithm 1).
**Algorithm 1**: Enumeration Algorithm**Input**: network topology *G*, the set of requests**Output**: The total cost $\varphi_{v}^{f}$, the number of deployed MEC servers *F*, and the set of deployment locations *W_opt_***Determining Feasible Deployment Sets**1: Initialize *U* denotes the set of all feasible deployment sets, *U*←*Φ*2: Compute lower limit (16)$$F^{lo}=\left\lceil \sum_{i}d_{i}/max\left(C•SC_{j}\right)\right\rceil$$3: **for**
*F*←*F^lo^* to *N+M*
**do**4: Use recursive algorithm to search sets *Q^F^*={ $Q_{1}^{f}$*,$Q_{2}^{f}$,..*$Q_{v}^{f}$} of MEC candidate locations which includes *F* MEC servers.5:  **for**
$Q_{v}^{f}$*∈*
*Q^F^*
**do**6:    **if** ∑*_j_*_∈$Q_{v}^{f}$_C•*SC_j_* <∑*_i_d_i_*
**then**7:       *Q^F^←Q^F^*-{$Q_{v}^{f}$}8:    **end if**9:   **end for**10: *U←U∪Q^F^*11: **end for****Calculating Total Cost of Feasible Deployment Sets**12: **for** each feasible set $Q_{v}^{f}$
*∈*
*U*
**do**13:  Calculate the minimum delay *T* of all users by invoking **Algorithm 2**14:  Calculate the deployment cost of unit workload with Equation (18)15:  Calculate the total cost $\varphi_{v}^{f}$*, $\varphi_{v}^{f}$=Dc+*Ψ•**(*T/|I|*)16: **end for**17: Optimal deployment set of MEC *W_opt_* ← select the set $Q_{v}^{f}$ with minimum total cost $\varphi_{v}^{f}$

**Step 2**: Calculate latency of all requests under a feasible deployment set. We aim to minimize the latency of all requests. In algorithm 2, first, we need to determine the priority of requests. The priority of requests is determined by using the request with minimum latency time first (MDTF) [30]. Assuming that there is only a request *i* in the network, the latency of request *i* to each MEC server in deployment set $Q_{v}^{f}$ is pre-calculated. The minimum latency of request *i* to each MEC server is selected as the latency of request *i*. We sequentially select a request from the sorted request set.

Then, we find the optimal MEC server for each request and calculate the corresponding latency of each request (lines 2–22 in algorithm (2). Since CU and DU have the master-slave characteristic in three-layer RAN architecture, the different coverage between the candidate locations of DU layer and the candidate locations of CU layer need to be considered. Therefore, the MEC server deployed at candidate location in the DU layer can only respond to requests in local zone, and the MEC server deployed at candidate location in the CU layer can respond to all requests in the network. Here, if the MEC server *j* belongs to DU layer and request *i* belongs to the local zone where location *j* is located, we need to calculate whether the total request arrival rate to MEC server *j* is greater than average service rate of MEC server *j* after request *i* is assigned to MEC server *j*. If average service rate *u_j_* of MEC server *j* is greater than total request arrival rate at MEC server *j,* the request *i* is assigned to MEC server *j* and *K* candidate paths is selected between DU *n* and MEC *j* by using K-shortest path (lines 3–7). All candidate paths *K_i_* are traversed for each request *i*, and the latency of request *i* for each candidate path *k* is calculated according to the following formulation (lines 8–11):

(17) $$t_{{}_{i,j}}^{k}=\nu \cdot \sum_{n}\sum_{r}Z_{i,r}\cdot Z_{r,n}\cdot d_{\left(r,n\right)}+\sum_{j}\left(\nu \cdot d_{i,(n,j)}^{k}+\frac{1}{u_{j}-\sum_{i}\lambda_{i}\cdot y_{i,j}}\right)\cdot y_{i,j}$$

The minimum $t_{i,j}^{k}$ is selected as the latency *t_i,j_* of request *i* assigned to MEC server *j* (line 12 in algorithm 2). If average service rate *u_j_* of MEC server *j* is less than the total request arrival rate at MEC server *j,* the latency *t_i,j_* of request *i* assigned to MEC server *j* is set as infinite (line 13 in algorithm 2), that is, the system stability cannot be satisfied. If the MEC server *j* belongs to CU layer, the lines 5–15 are repeated and the latency *t_i,j_* of request *i* is calculated (lines 18–20 in algorithm 2).

Finally, all MEC servers in the set $Q_{v}^{f}$ are traversed for each request *i*, and the latency *t_i,j_* of request *i* assigned to each MEC server *j* is calculated. The minimum *t_i,j_* is selected as the latency *t_i_* of request *i*, and the MEC server *j* with the minimum *t_i,j_* as the optimal MEC server of request *i* (lines 21–22 in algorithm 2). We traverse all requests and calculate the latency of all requests (line 23 in algorithm 2).
**Algorithm 2:** Assign Requests to MEC Servers**Input**: network topology *G*, the set of requests**Output**: the total delay of all request *T*.1:**while** request *i*
*∈ I* in ascending order of delay **do**2:  **for** MEC server *j*
*∈*
$Q_{v}^{f}$
**do**3:   **if**
*j ∈* N then4:    **if** request *i* belongs to the local zone where *j* is located5:     **if** C•*SC_j_*>∑*_i_d_i_* •*y_i,j_*
**and**
*u_j_* >∑*_i_*
***λ_i_*** •*y_i,j_*
**then**6:      Obtain RRU *r* that request *i* belongs to and RRU *r* being connected with DU *n*7:      Pre-calculate *K* candidate paths between DU *n* and MEC *j* by using *K*-shortest path8:        **for** each path *k ∈ K_i_*
**do**9:        Allocation continuity wavelength with First-Fit on path *k*10:        Calculate the delay based on Equation (17)11:       **end for**12:     *t_i,j_*← min *_k_*
$t_{i,j}^{k}$13:      **else**
*t_i,j_* ← ∞14:      **end if**15:     **else**
*t_i,j_* ← ∞16:    **end if**17:   **end if**18:  **if**
*j ∈ M*
**then**19:   Repeat lines 5-1320:  **end if**20: **end for**21:  *t_i_* ← min *_j_ t_i,j_*22:  Find the optimal MEC j* with minimize delay,     i.e., j* = arg min *_j__∈$q_{v}^{k}$_*(*t_i,j_*)23: *T*←*T*+*t_i_*24: Update network status25: **end while**

**Step 3**: Calculate total cost of each feasible deployment set. We first calculate the latency of all requests under a feasible candidate deployment set according to algorithm 2. Then, we calculate the MEC servers’ deployment cost of the corresponding deployment set according to Equation (18). Finally, we traverse all feasible deployment sets, and calculate the sum of deployment cost and latency cost for each feasible deployment set. We choose the deployment set that has the minimum deployment cost and latency cost as the optimal deployment scheme.

(18) $$Dc=\sum_{j\in Q_{v}^{k}}\frac{f_{j}+P\cdot SC_{j}}{C\cdot SC_{j}}$$

**Theorem** **1.** *The computational complexity of enumeration algorithm is O(|N+M${|\cdot C}_{|N+M|}^{|R|}$·|I|·|R|·K).*

**Proof.**  In *Algorithm* 1, lines 3–11 find all candidate deployment sets under different numbers of MEC servers, whose computational complexity is *O*(|*N*+*M*${|\cdot C}_{|N+M|}^{|R|}$), where *N*+*M* is the number of candidate locations, *R* is the number of MEC servers. Then for each deployment set, the requests allocation in Algorithm 2 will be executed, whose computational complexity is *O*(|*I*|·|*R*|·*K*), where *I* is the number of requests, *K* is the number of candidate paths. Therefore, the overall computational complexity of enumeration algorithm is *O*(|*N*+*M*${|\cdot C}_{|N+M|}^{|R|}$·|*I*|·|*R*|·*K*). □

### 5.2. Approximate Algorithm based on the improved entropy weight and TOPSIS method

We propose an approximate algorithm for solving MEC servers’ deployment problem quickly and effectively, which combines entropy weight [17,31], and TOPSIS method [32] based on unassigned requests ratio. As shown in Algorithm 3, first, we determine the indicators of each candidate location. Then, based on the improved entropy weight and TOPSIS method, we evaluate the score of each candidate location. Finally, we will get *K* most suitable locations to be co-located with MEC servers. The detailed processes are shown in the following steps.
**Algorithm 3:** Approximate Algorithm based on the improved entropy weight and TOPSIS method**Input**: network topology *G*, the set of requests**Output**: The total cost, the number of deployed MEC *F*, and the set of deployment locations *W_opt_*.1: Initialize *W_opt_*←*Φ, T*←*Φ, K=*02: Calculate the deployment cost of unit workload for each candidate location with Equation (19)3: Execute **Algorithm 4** to calculate average delay of each candidate location4: Evaluate *c_j_* according to Equation (28)5: **for** 0 to *R*
**do**6:  **if**
*I*≠Φ **then**7:    **for**
*j*
*∈* (*N+M*)/*W_opt_*
**do**8:    Calculate unassigned requests U*I_j_*9:    Calculate *S_j_* according to Equation (29)10:   **end for**11:   Sort *S_j_* in decreasing order12:   Find the first *S_j_*13:   *W_opt_*←*W_opt_* ∪ {*j*}14:   Determine the unassigned requests I by invoking **Procedure 1**.15:   *R*=*R*+116:   Update the network state17:  **else** exit18:  **end if**19: **end for**20: Calculate the deployment cost of unit workload for each MEC in the set *W_opt_* with Equation (18)21: Calculate the total delay *T* of all requests by executing **Algorithm 2**22: Calculate the total cost $\varphi_{v}^{f}$*, $\varphi_{v}^{f}$=Dc+*Ψ*•*(*T/|I|*)

**Step 1**: Determine the indicators for each candidate location. There are many complex factors affecting on location decision-making of MEC server, such as latency, bandwidth, energy consumption, and cost. In the location selection phase of MEC server, deployment cost and latency are the main considerations [13]. We first calculate the deployment cost of the unit workload of each candidate location according to Equation (19) in line 2.

(19) $$Dc_{j}=\frac{f_{j}+P\cdot SC_{j}}{C\cdot SC_{j}}$$

Then, we calculate the average latency of requests served at each candidate location according to Algorithm 4. If the candidate location *j* belongs to DU layer, the requests in local zone where location *j* is located are sorted in ascending order of latency (lines 4–5 in algorithm 4). The sorted requests are assigned to candidate location one by one until the capacity reaches the capacity of MEC server at candidate location or the total request arrival rate is bigger than average service rate of MEC server at the candidate location (lines 6 in algorithm 4). The latency $t_{i,j}^{k}$ of request *i* for each candidate path *k* is calculated according to Equation (17) and the minimum $t_{i,j}^{k}$ is selected as the latency *t_i,j_* of request *i* assigned to MEC server *j* (lines 8-9 in algorithm 4). The average latency of location *j* belonging to the DU layer is calculated in line 15. If the candidate location belongs to CU layer, we should calculate latency of each request in the network. The lines 6–15 in algorithm 4 are repeated, and the average latency of location *j* belonging to the CU layer is calculated (lines 17–20 in algorithm 4).
**Algorithm 4:** Calculate Average Delay for Each Candidate Location**Input**: network topology *G*, the set of user requests**Output**: The average delay of requests to each candidate location *aveT_j_*.1: **for**
*j*
*∈ J*
**do**2:   Initialize *λ_j_*←Φ, *D*←Φ, *R*←Φ, *T_j_*←Φ3:    **if**
*j ∈ N*
**then**4:     Pre-calculate delay of each request in local zone where location *j* is located5:     **for** request *i* in the zone according to ascending order of delay **do**6:        **if** C•*SC_j_*>∑*_i_d_i_* •*y_i,j_*
**and**
*u_j_* >∑*_i_*
***λ_i_*** •*y_i,j_*
**then**7:         Allocate the request *i* to MEC *j*8:         Calculate the delay of request *i* for each candidate path *k* with Equation (17)9:          *t_i,j_*← *min _k_*
$t_{i,j}^{k}$10:          *T_j_=T_j_+t_i,j_*11:          *R=R*+112:        **else** exit13:      **end if**14:    **end for**15:    *aveT_j_*= *T_j_/R*16:   **end if**17:   **if**
*j ∈ M*
**then**18:    Pre-calculate delay of each request in the network19:    Repeat lines 6-15, calculate the *aveT_j_*
20:   **end if**21: **end for**

**Step 2**: Calculate the score of each candidate location. The indicators of the candidate locations have been determined by using the above step.

First, the entropy of each indicator is calculated by using entropy weight method. The deployment cost and average latency are cost type indicators, which represents that the smaller the attribute value reaches, the better the location is. The standardization of each indicator at each candidate location is as follows:

(20) $${x^{\prime }}_{j\sigma }=\frac{\underset{\sigma }{\max}(x_{j\sigma })-x_{j\sigma }}{\underset{\sigma }{\max}(x_{j\sigma })-\underset{\sigma }{\min}(x_{j\sigma })}$$

where *x_jσ_* is the original value of indicator *σ* at location *j*; max(*x_jσ_*) is the maximum value of indicator *σ*; min(*x_jσ_*) is the minimum value of indicator *σ*.

To calculate the index value proportion $x_{j\sigma }^{\prime }$ of the *σth* indicator of the *jth* location, the formula is defined as:

(21) $$\varsigma_{j\sigma }=\frac{x_{j\sigma }^{\prime }}{\sum_{j}x_{j\sigma }^{\prime }}$$

We evaluate the input entropy of each indicator and the entropy weight of each indicator according to Equations (22) and (23), respectively:

(22) $$E_{\sigma }=-\frac{1}{\ln(N+M)}\sum_{j}\varsigma_{j\sigma }\ln\varsigma_{j\sigma }$$

(23) $$w_{\sigma }=\frac{1-E_{\sigma }}{\sum_{\sigma }\left(1-E_{\sigma }\right)}$$

Then, the score of each candidate location is calculated by combining entropy weight and the TOPSIS method. To eliminate the influence of indicator dimension and its variation range on evaluation results, the original matrix is normalized as follows:

(24) $$z_{j\sigma }=\frac{x_{j\sigma }}{\sqrt{\sum_{j}x_{j\sigma }^{2}}}$$

The weighted normalized decision matrix is calculated as:

(25) $$V={\left(v_{j\sigma }\right)}_{m\times n}={\left(\eta_{\sigma }\cdot w_{\sigma }\cdot z_{j\sigma }\right)}_{m\times n}$$

The Euclidean distance of each candidate location from the ideal location and the negative-ideal location is calculated by Equations (26) and (27), respectively.

(26) $$d_{j}^{+}=\sqrt{\sum_{\sigma }{\left(v_{j\sigma }-v_{\sigma }^{+}\right)}^{2}}$$

(27) $$d_{j}^{-}=\sqrt{\sum_{\sigma }{\left(v_{j\sigma }-v_{\sigma }^{-}\right)}^{2}}$$

where $v_{\sigma }^{+}$ and $v_{\sigma }^{-}$ are positive ideal location and negative-ideal location.

The score of each candidate location is formulated as the relative closeness to the ideal solution:

(28) $$c_{j}=\frac{d_{j}^{-}}{d_{j}^{+}+d_{j}^{-}}$$

Finally, on the one hand, to avoid the MEC servers gather around in the same area, and on the other hand, to prevent a large capacity MEC server only respond to a small number of unassigned requests, we consider the influence of unassigned requests on deployment locations. We introduce the metric of “unassigned requests ratio”. The *UI_j_* is the number of unassigned requests at candidate location *j*, and *TI_j_* is the total of requests at candidate location *j*. Let “*UI_j/_TI_j_*” denote the unassigned requests ratio. When the unassigned requests ratio is high, this means that candidate location *j* has more unassigned requests.

Therefore, the candidate location *j* is more suitable to be co-located with MEC server. Considering the influence from other already deployed MEC servers and unassigned requests, the score of each candidate location can be re-evaluated by:

(29) $$S_{j}=\left\{ \begin{array}{l} c_{j},\;W_{opt}=\Phi \\ \frac{UI_{j}}{TI_{j}}\cdot c_{j},\;W_{opt}\neq \Phi \end{array}\right.$$

**Figure 3:**
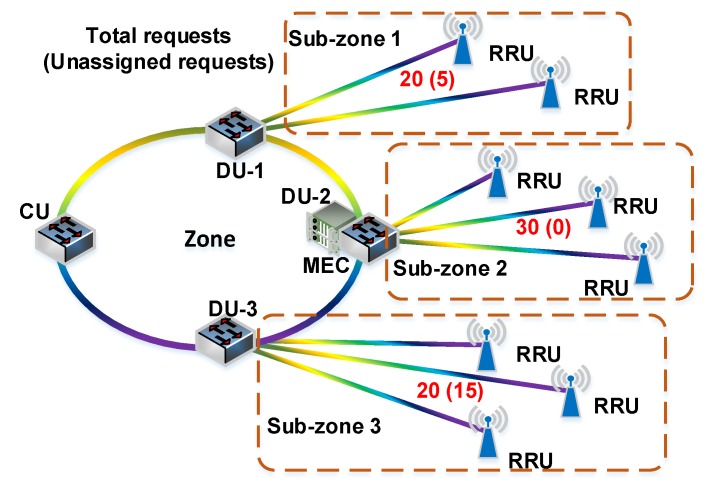
An example of determining the deployment location.

Taking Figure 3 as an example, the location of DU-2 has already deployed MEC server. Suppose that all requests in sub-zone 2 and some requests in sub-zone 1 and sub-zone 3 have been assigned to the MEC server that is co-located with DU-2. The total number of requests of sub-zone 1, sub-zone 2 and sub-zone 3 are 20, 30 and 20, respectively. The unassigned requests of sub-zone 1 and sub-zone 3 are 5 and 15, respectively. To meet the demands of unassigned requests, the next deployment location needs to be determined. Suppose that values of *c_j_* of DU-1, DU-3, and CU are 0.45, 0.47, and 0.54, respectively. The location co-located with CU is the optimal deployment location. Considering the influence from unassigned requests, the score *S_j_* of DU-1, DU-3, and CU are 0.11, 0.35, and 0.15, respectively ((5/20) × 0.45 = 0.11, (15/20) × 0.47 = 0.35, (20/70) × 0.54 = 0.15). Therefore, the location co-located with DU-3 is the optimal deployment location. Compared to deploying the MEC server at location co-located with CU, the MEC server deployed at location co-located with DU-3 can save resources based on meeting the request requirements.

**Step 3**: Find near optimal deployment set and calculate total cost. First, we determine whether there are unassigned requests (line 6 in algorithm 3). If there are unassigned requests, a candidate location with the highest score *S_j_* is selected (lines 7–12 in algorithm 3). We will allocate requests to the MEC server *j* according to ascending order of latency and determine the unassigned requests (Procedure 1). The system updates the network and unassigned requests are included in *I* (line 16 in algorithm 3). Then, we repeat the above process until all requests are assigned, and obtain the near optimal deployment set. Finally, the sum of deployment cost and latency cost is calculated for near optimal deployment set (lines 20–22 in algorithm 3).
**Procedure 1:** Determine Unassigned Requests**Input**: network topology *G*, the set of requests**Output**: unassigned requests *I*1: **if**
*j ∈ N*
**then**2:   Pre-calculate delay of each request in the zone where location *j* is located3:   **for** each *i* in the zone according to ascending order of delay **do**4:    **if** C•*SC_j_*>∑*_i_d_i_* •*y_i,j_*
**and**
*u_j_* >∑*_i_*
***λ_i_*** •*y_i,j_*
**then**5:        Allocate the request *i* to MEC *j*6:        *I←I*/*i*7:      **else** exit8:    **end if**9:   **end for**10:   **if**
*j ∈ M*
**then**11:   Calculate delay of each request in the network12:   Repeat the above process, calculate *I*13:  **end if**14: **end if**

**Theorem** **2.** *The computational complexity of approximate algorithm is O(|I|·|N+M|·(K+|R|)).*

**Proof.**  In *Algorithm 3*, we first evaluate the indicators for each candidate location. The line 2 evaluate the deployment cost of unit workload for each candidate location, whose computational complexity is *O*(|*N*+*M*|). The average latency of each candidate location will be evaluated by Algorithm 4, whose computational complexity is *O*(|*I*|·|*N*+*M*|·*K*). Then, we find *R* optimal locations to co-located with MEC servers iteratively in lines 5–19. In each iteration, we need to determine unassigned requests in Procedure 1 with *O*(|*I*|). Thus, the computation complexity of lines 5–19 is *O*(|*N*+*M*|·|*I*|·|*R*|). As a result, the overall computational complexity of approximate algorithm is *O*(|*I*|·|*N*+*M*|·(*K*+|*R*|)). □

## 6. Simulation Results and Discussion

**Figure 4:**
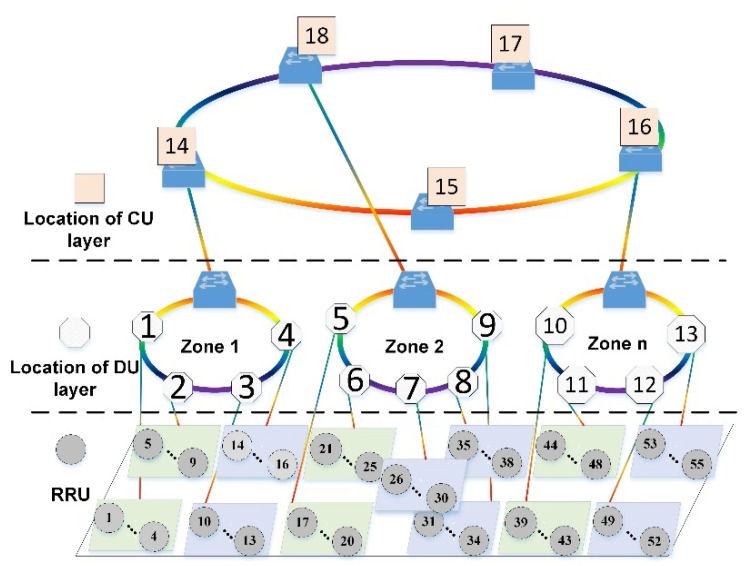
Simulation network topology.

In this section, we evaluate the performance of the proposed algorithms. We consider a simulation network topology as shown in Figure 4, which consists of 55 RRUs, 13 candidate locations for MEC servers at DU layer, and five candidate locations for MEC servers at CU layer. We set the following parameters as in [28]. The capacity of MEC server at DU layer is uniformly distributed within [5,10] × 10^4^ cycles. The capacity of MEC server at CU layer is uniformly distributed within [1,5] × 10^5^ cycles. The average service of MEC server is proportional to the capacity of MEC server. The average service *u_j_* of each MEC server deployed at DU layer is chosen according to the Normal distribution with an average of 800 and a variance of 20, i.e., N(800, 20). The average service *u_j_* of each MEC server deployed at CU layer is chosen according to the Normal distribution with an average of 2000 and a variance of 100, i.e., N(2000, 100). We set the length of link in the simulation network topology as in [33]: the length of RRU-DU, DU-DU, DU-CU, and CU-CU are uniformly distributed with [1,9] km, [40,80] km, [80,100] km, and [100,200] km, respectively. We assume that the number of wavelengths per fiber is 80 [34]. Considering that backhaul link is shared by more requests than mid-haul and fronthaul, the number of fiber pairs of the backhaul link between CUs is six. Similarly, each mid-haul link from DU to CU has four fibers pairs, each mid-haul link from DU to DU has two fibers pairs, and each fronthaul link from RRU to DU has one fiber pairs. Meanwhile, 800 requests are uniformly distributed among the RRUs. The average size of requests is chosen according to the Normal distribution with an average of 500 cycles and a variance of 50 cycles, i.e., N(500, 50) cycles. Service requests follow Poisson distribution, we randomly choose the average generation rate of requests between 0 and λ. We set λ = 1.7 request/s.

We analyzed the proposed algorithm from two perspectives, from the perspective of the total cost, we start the numerical analysis by contrasting enumeration algorithm and approximate algorithm against Heaviest-Location First Algorithm (HLFA) [18] and Latency-based Algorithm (LBA) [35]. For HLFA, it deploys MEC servers at the candidate locations having the heaviest requests. The LBA is to minimize the latency between requests and MEC servers serving the requests. From the perspective of the relationship between the deployment cost of service providers and latency of requests, we start the numerical analysis by contrasting the approximate algorithm against HLFA and LBA. In this simulation, we mainly evaluated two aspects of performance: deployment cost and latency. The deployment cost reflects economic benefits, which is an important property for service providers. The latency reflects the quality of the user experience, which is an important property for users to consider.

### 6.1. Performance of Enumeration Algorithm and Approximate Algorithm in Terms of Total Cost

**Figure 5:**
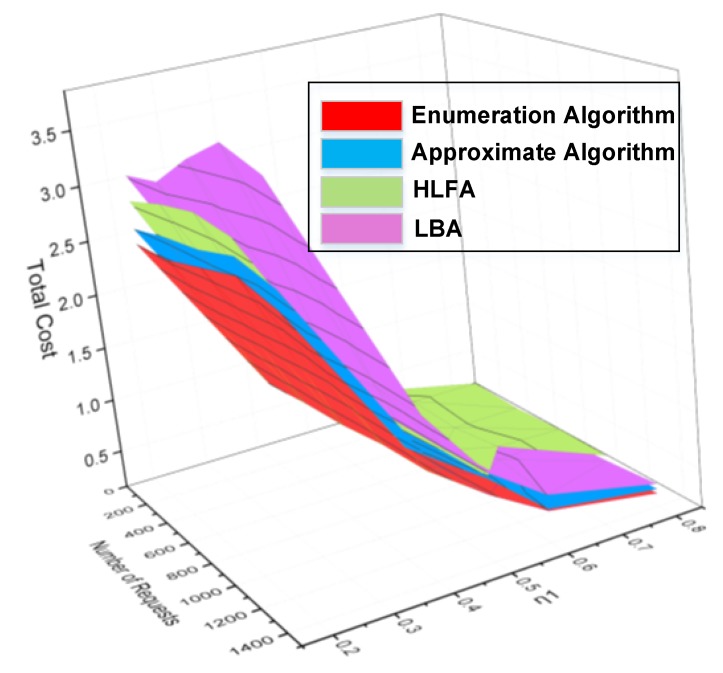
Comparisons of total cost under different number of requests and different tradeoff coefficient.

**Figure 6:**
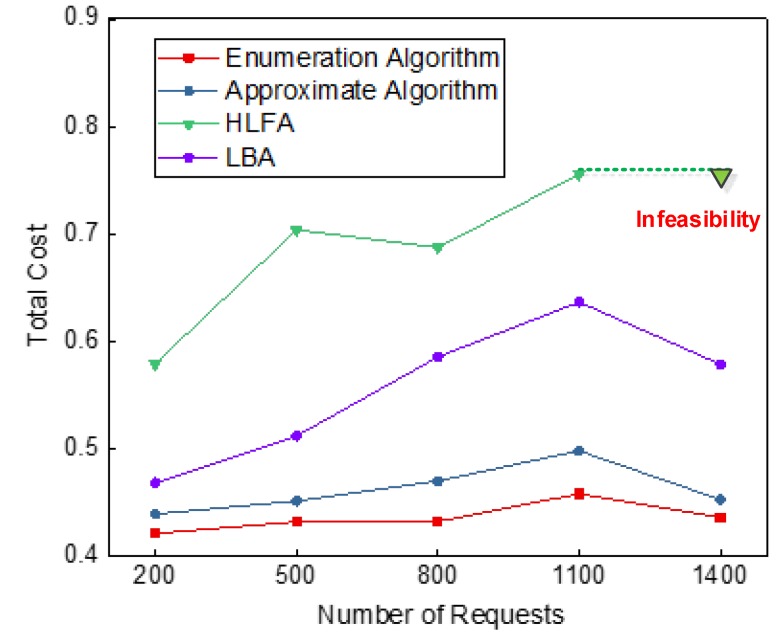
Comparisons of total cost under different number of requests, when *η*_1_ = 0.6.

**Figure 7:**
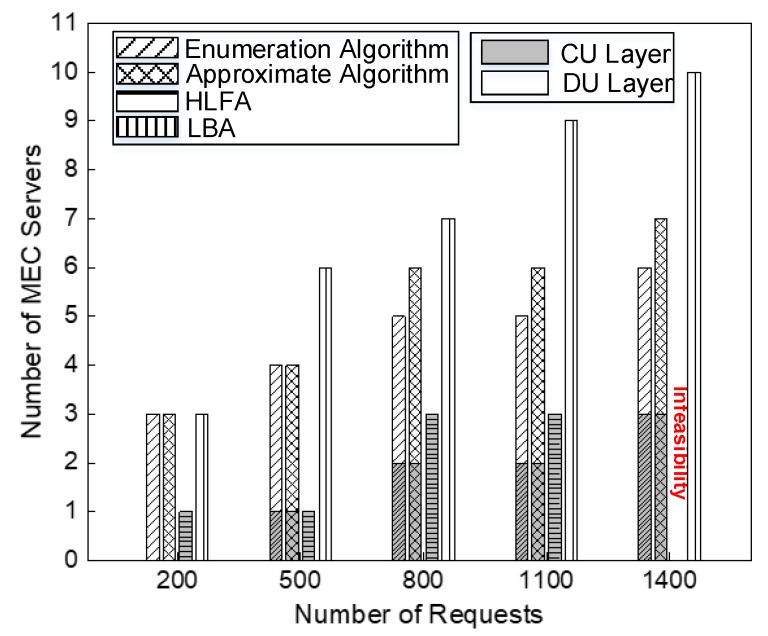
Comparisons of the number of MEC servers under different number of requests, when *η*_1_ = 0.6.

**Figure 8:**
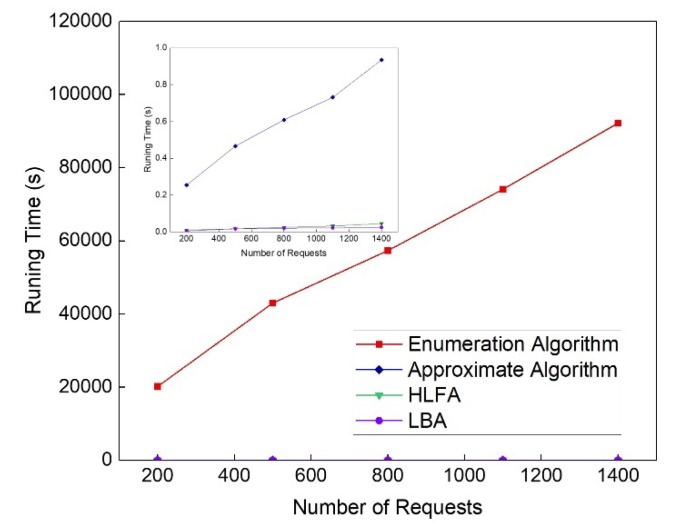
Comparisons of the running time.

A good deployment scheme not only ensures low average latency of requests, but also needs to consider the deployment cost of service providers. Figure 5, Figure 6, Figure 7 and Figure 8 show the simulation results of the performance of enumeration algorithm, approximate algorithm, HLFA, and LBA with different number of requests. Based on this figure, we made the following observations.

Figure 5 shows the total cost of different deployment scheme under different number of requests and different tradeoff coefficient *η*_1_. First, it is clearly observed from Figure 5 that enumeration algorithm can get the optimal solution, while approximate algorithm can obtain a near-optimal solution better than that of HLFA and LBA. The reason is that the enumeration algorithm and approximate algorithm minimize both deployment cost and latency. When deployment cost increases, the average latency of requests may reduce. HLFA focuses on the deployment cost, which leads to large average latency of requests. LBA focuses on the average latency of requests, which leads to high deployment cost. Result shows that the deployment cost and latency are important factors in solving MEC servers’ deployment problems.

Take the tradeoff coefficient *η*_1_ is set to 0.6 as an example, as shown in Figure 6, we further find that when the tradeoff coefficient *η_1_* is 0.6, LBA always outperforms HLFA. This is because when *η*_1_ equals 0.6, the dominant cost is the average latency cost. The average latency of HLFA is greater than the average latency of LBA. Therefore, the total cost of HLFA is greater than the total cost of LBA. Note that, for 1400 requests, no feasible deployment scheme is found by using HLFA. This is because the MEC servers deployed by using HLFA are all located at CU layer, which is far from the requests. When assigning requests, a large number of wavelengths need to be occupied, resulting in insufficient wavelength resources in C-RAN.

Secondly, we observe that the enumeration algorithm and approximate algorithm keep relatively stable performance with different numbers of requests in Figure 6. The stable performance of enumeration algorithm and approximate algorithm comes from its combination of deployment cost and latency. As shown in Figure 7, as the number of requests increases, the number of MEC servers will increase, resulting in increased deployment cost. Meanwhile, more MEC servers in the network can provide more computing resources for those requests, which leads to less processing latency. Therefore, the enumeration algorithm and approximate algorithm keep relatively stable performance.

Thirdly, as further shown in Figure 6, we also observed the total costs of the enumeration algorithm and the approximate algorithm fluctuate according to the number of requests. This is because the total cost is affected by both deployment cost and latency. As shown in Figure 7, when the number of requests increases from 800 to 1100, the number of MEC servers does not change. However, as the number of requests increases, the average computing latency of requests increases. Therefore, the total cost is increased when the number of requests increases from 800 to 1100. When the number of requests increase from 1100 to 1400, the number of MEC servers increases. The average computing latency of requests is significantly reduced. Therefore, the total cost is reduced when the number of requests increases from 1100 to 1400.

Figure 8 shows the running time of the algorithms. We observe that all algorithms have increasing running time with the number of requests increasing. The running time of the enumeration algorithm dominates the others as they find the total cost of all deployment sets, which is time consuming. approximate algorithm can get much lower complexity compared with the enumeration algorithm. Though the running time of HLFA and LBA is lower than the approximate algorithm, approximate algorithm has the closest performance to that of the enumeration algorithm on minimizing the total cost.

### 6.2. Performance of Approximate Algorithm in Terms of Deployment Cost and Average Latency

**Figure 9:**
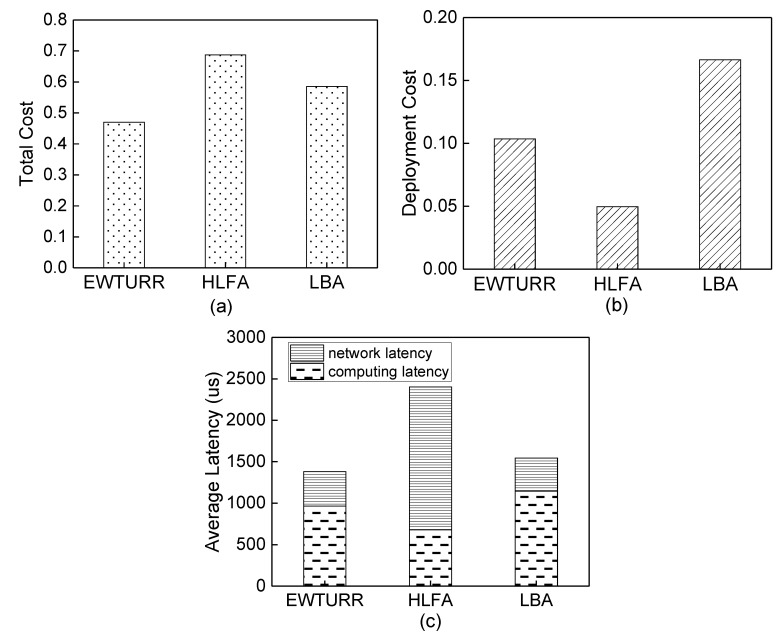
Performance illustration of approximate algorithm, heaviest-location first algorithm (HLFA) and latency-based algorithm (LBA) (**a**) Comparisons of total cost under different algorithms. (**b**) Comparisons of deployment cost under different algorithms. (**c**) Comparisons of average latency under different algorithms.

The main goal of the simulation is to illustrate the impact of the deployment cost and latency on the total cost. We fixed the number of requests as 800. Figure 9a shows the total cost, in which the approximate algorithm achieves lower total cost as compared to the other two algorithms. The reason is that the approximate algorithm combines deployment cost and latency. As shown in Figure 9b, the result indicates that the approximate algorithm decreases 37.8% deployment cost as compared to the LBA. This is because LBA chooses to deploy MEC servers in candidate locations closest to requests without considering the deployment cost of each candidate location. That is, LBA always selects the candidate locations of DU layer to deploy the MEC servers. Therefore, LBA causes high deployment cost when serving the same number of requests due to the limited computing and storage capacity and high infrastructure cost of MEC servers deployed at the DU layer.

Figure 9c shows the average latency of requests by applying different deployment algorithms of MEC servers. We can see that the average latency of the approximate algorithm is decreased by 42.6% as compared to HLFA, and 10.6% as compared to LBA. The reason is that the approximate algorithm minimizes both the network latency and computing latency. As further shown in Figure 9c, we observe that the approximate algorithm balances network latency and computing latency. In Figure 7, the optimal deployment locations of MEC servers found by HLFA are all located at CU layer, which has maximum network latency because of the farther locations of MEC servers deployed at CU layer and minimum computing latency because of large computing capacity of MEC servers deployed at CU layer. The optimal deployment locations of MEC servers found by LBA is located at DU layer, which has maximum computing latency because of the limited computing capacity of MEC servers deployed at DU layer and minimum network latency because of the proximity of requests. The optimal deployment locations of MEC servers found by approximate algorithm include locations of DU layer and CU layer. Therefore, approximate algorithm can weigh network latency and computing latency.

**Figure 10:**
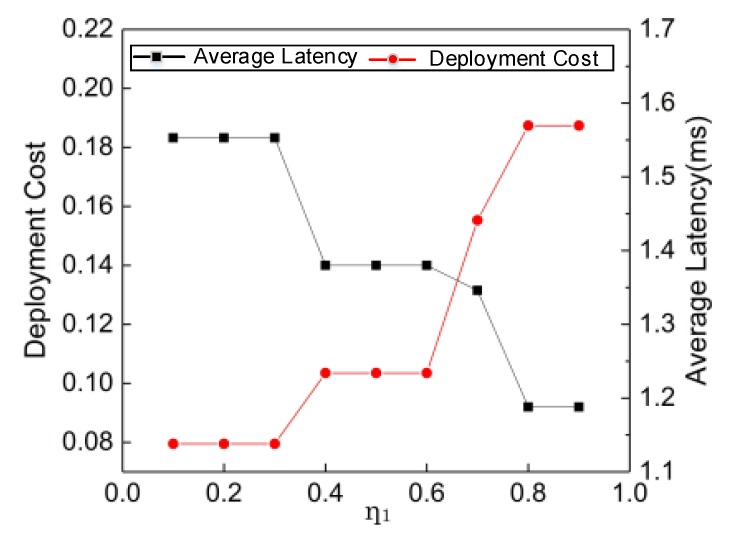
Impact of *η*_1_ on deployment cost and average latency.

Figure 10 illustrates the deployment cost and average latency for approximate algorithm with different *η*_1_. When *η*_1_ increases, approximate algorithm starts to pay more attention to the average latency. As a result, it tends to deploy more MEC servers and select optimal location at DU layer to reduce the average latency while sacrificing deployment cost. Therefore, we can just adjust the relation between deployment cost and average latency by selecting a suitable *η*_1_ according to the actual requirement of the network.

## 7. Conclusions

In this paper, we have introduced a novel hierarchical deployment of MEC servers’ optimization strategy in C-RAN over WDM ring networks to minimize deployment cost and average latency. We have proposed enumeration algorithm and approximate algorithm to solve MEC servers’ deployment and user allocation problem. Simulation results showed that the proposed approximate algorithm based on the improved entropy weight and TOPSIS method can obtain lower total cost comparing the HLFA and LBA. Additionally, the hierarchical deployment of MEC servers could tradeoff deployment cost and latency. The proposed algorithms will serve as a theoretical foundation for further MEC server network planning.

As a future work, we are currently developing a joint MEC servers’ deployment and content caching (i.e., video) from the perspective of content providing. By analyzing the different video attributes (location, size, popularity), we determined the optimal locations of MEC servers in the network, and selected the MEC servers to cache videos.

## Figures and Tables

**Table 1:** Summary of Notations.

Notation	Definition
*E*	The set of fiber links in the network, index *e*.
*I*	The set of requests, index *i*.
*R*	The set of RRUs.
*N*	The set of DUs.
*M*	The set of central units.
*J*	The set of candidate locations of MEC servers.
(*l*, *m*)	The link (*l*, *m*) between node *l* and node *m* (*l, m∈* *J*, (*l*, *m*)*∈* *E*).
*f_j_*	The cost of rental site.
*SC_j_*	The number of physical machines.
*d_i_*	The computing resource demand of request *i*
*L_l,m_*	The distance between node *l* and node *m*.
*u_j_*	The average service rate of MEC server *j*.
*λ_i_*	The average generation rate of request *i*.
*Z_i,r_*	Binary indicator, which denotes request *i* in the coverage area of RRU *r*.
*Z_r,n_*	Binary indicator, which denotes RRU *r* connects with DU *n*.
*Z_i,n_*:	Binary indicator, which denotes request *i* in the coverage area of DU *n*.
*P*	The price of a physical machine.
*C*	Computing capacity of a physical machine
*W*	Maximum number of wavelengths available at each link.
*ʋ*	Propagation time of unit distance.
*x_j_*	Binary variable, represents whether a MEC server is placed at candidate location *j* (i.e., *x_j_* = 1) or not (i.e., *x_j_* = 0).
*y_i,j_*	Binary variable, represents whether request *i* is handled by MEC server *j* (i.e., *y_i,j_* = 1) or not (i.e., *y_i,j_* = 0).
$Q_{i,n,j}^{(l,m),w}$	Binary variable, indicates that request *i* uses *w_th_* wavelength on link (*l, m*) when request *i* within the coverage area of DU *n* is handled by MEC server *j*.
$Q_{i,n,j}^{w}$	Binary variable, indicates that request *i* uses *w_th_* wavelength when request *i* within the coverage area of DU *n* is handled by MEC server *j*.
